# Dominant Glint Based Prey Localization in Horseshoe Bats: A Possible Strategy for Noise Rejection

**DOI:** 10.1371/journal.pcbi.1002268

**Published:** 2011-12-01

**Authors:** Dieter Vanderelst, Jonas Reijniers, Uwe Firzlaff, Herbert Peremans

**Affiliations:** 1 Active Perception Lab, University Antwerp, Antwerp, Belgium; 2 Department of Biology, University Antwerp, Antwerp, Belgium; 3 Lehrstuhl für Zoologie, Technical University München, Freising-Weihenstephan, Germany; Indiana University, United States of America

## Abstract

Rhinolophidae or Horseshoe bats emit long and narrowband calls. Fluttering insect prey generates echoes in which amplitude and frequency shifts are present, i.e. glints. These glints are reliable cues about the presence of prey and also encode certain properties of the prey. In this paper, we propose that these glints, i.e. the dominant glints, are also reliable signals upon which to base prey localization. In contrast to the spectral cues used by many other bats, the localization cues in Rhinolophidae are most likely provided by self-induced amplitude modulations generated by pinnae movement. Amplitude variations in the echo not introduced by the moving pinnae can be considered as noise interfering with the localization process. The amplitude of the dominant glints is very stable. Therefore, these parts of the echoes contain very little noise. However, using only the dominant glints potentially comes at a cost. Depending on the flutter rate of the insect, a limited number of dominant glints will be present in each echo giving the bat a limited number of sample points on which to base localization. We evaluate the feasibility of a strategy under which Rhinolophidae use only dominant glints. We use a computational model of the echolocation task faced by Rhinolophidae. Our model includes the spatial filtering of the echoes by the morphology of the sonar apparatus of *Rhinolophus rouxii* as well as the amplitude modulations introduced by pinnae movements. Using this model, we evaluate whether the dominant glints provide Rhinolophidae with enough information to perform localization. Our simulations show that Rhinolophidae can use dominant glints in the echoes as carriers for self-induced amplitude modulations serving as localization cues. In particular, it is shown that the reduction in noise achieved by using only the dominant glints outweighs the information loss that occurs by sampling the echo.

## Author Summary

Rhinolophidae are echolocating bats that hunt among vegetation. The foliage returns clutter echoes that potentially mask the echoes of insect prey. However, prey introduces frequency and amplitude shifts, called glints, into the echo to which these bats are highly sensitive. Therefore, these glints are used by Rhinolophidae to detect prey and infer its properties. One of the defining characteristic of consecutive dominant glints is that they have a very stable amplitude. This is, consecutive wing beats of the insect produce dominant glints with more of less the same amplitude. Owing to the strategy Rhinolophidae use to locate prey, the stable amplitude of glints makes these parts of the echoes ideal signals to use to locate prey. In this paper, we demonstrate the feasibility of strategy under which Rhinolophidae use only the dominant glints in the echo for locating prey.

## Introduction

Rhinolophidae or Horseshoe bats, a family of echolocating bats, hunt for fluttering insects amongst vegetation [1], [2]. This implies that, with each call, they receive a large number of echoes most of which originate from foliage. They have evolved an echolocation system that allows detecting prey under these difficult circumstances by encoding the presence and the properties of prey by frequency and amplitude modulations in the returning echo (reviewed in [3]).

Rhinolophidae emit long narrowband pulses where most energy is contained in a single and well-controlled frequency component. Fluttering prey introduces frequency and amplitude modulations into the returning echo called glints [4]–[7]. Glints reliably signal the presence of prey to the bat. Indeed, Rhinolophidae only pursue insect prey that introduces glints in the echoes [3], [7], [8]. In addition to merely signalling the presence of prey, it has been argued that the glints provide Rhinolophidae with cues about a number of prey properties (reviewed in refs. [3], [7], [9]). The prey property encoded in the glints that is best studied is the wing beat frequency. The wing beat frequency of an insect can readily be inferred from an echo by counting the glints. In experiments, Rhinolophidae were able to discriminate between targets fluttering at different rates, e.g. [5], [7], [8].

For the localization of echoes in azimuth and elevation bats using broadband calls depend on spectral cues created by the transfer function of the outer ears (e.g. [10]–[12]). The use of a narrow frequency band to perform echolocation prevents Rhinolophidae from using spectral cues to localize reflectors in space [13]. To overcome this, they vigorously move their ears while echolocating [13]–[17]. The movement of the pinnae (which they coordinate with the reception of the echo) imposes amplitude modulations upon the incoming echo. The exact modulation patterns depend on the reflector position (azimuth and elevation). As it has been shown that these amplitude cues provide stable localization information [18], [19], it is assumed that this cue is also used by the bat to estimate the origin of the echo [13]. Indeed, when Rhinolophidae are prevented from moving their pinnae, their ability to locate obstacles deteriorates [14], [20].

In the current paper, we present simulations showing that glints carry the most reliable information for prey localization (as has been shown to be the case for prey classification,). Only fluttering insect prey produces glints. Therefore, the amplitude of glints is not influenced by interfering echoes from the background vegetation. In contrast, the carrier frequency band will contain a number of overlapping echoes from foliage resulting in spurious amplitude variations that are not due to pinna movement [18]. By only processing the glints when determining the location of prey, the bat could effectively reduce the influence of clutter on the localization cues. Fluttering prey not only introduces frequency shifts into the echo but also considerable amplitude variations [4], . However, the amplitude of the dominant glint is rather stable. Dominant glints can be defined as the most Doppler shifted parts in the echo and are produced at the instant the insects wings are perpendicular to the impinging sound waves [4]. In ensonification experiments, the amplitude of the dominant glint was found to have a standard deviation of less than 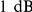 [4] while the amplitude of the echo across its entire duration can fluctuate by up to 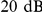 [3]. In sum, for localization, *Rhinolophidae* could substantially reduce the noise (i.e. unknown amplitude variations) by focussing on the dominant glints. This would reduce both the interference by echoes from foliage and stabilize the glint amplitude.

**Figure 1:**
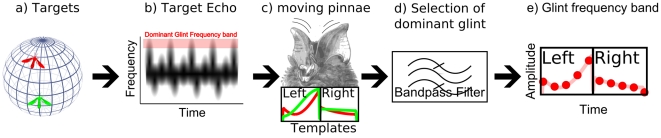
Illustration of the origin of the echolocation cues and the sampling that would occur by focusing on the glints in Rhinolophidae. Fluttering insects in the environment (a) cause echoes containing Doppler shifted glints. (c) The moving pinnae of *R. rouxii* impose an amplitude modulation upon the received echo. This modulation is different for each position of the target with respect to the bat. The echoes of the red and green insects would be modulated differently. (d) The dominant glints are selected by attending the most Doppler shifted parts of the echo. (e) This results in a sampled template (location of the red dots) at the left and the right ear (illustrated for the red insect). It should be noted that the spectrogram shown in (b) and the templates shown in (c) are stylized versions of a real echo containing glints and templates respectively. See [4], [5], [8] for real examples of echoes from fluttering insects and figure 7 for real templates.

Focussing on the dominant glint potentially comes at a cost. Depending on the flutter rate of a target, only a limited number of dominant glints will be present in each echo. By only processing these, the bat would effectively use a sampled version of the echo where information is only available at discrete times of dominant glints caused by the wing beat. This process is illustrated in figure 1. Using a sampled version of the echo, potentially reduces the amount of localization information generated by the moving ears that is transferred to higher auditory centres. Indeed, unless the information generated by the amplitude modulations is robust against being sampled at a low rate (given by the dominant glint rate), the clutter rejection mechanism would pose a limit to the echolocation capacity of the animal.

In this paper, we use a computational model of the echolocation task faced by Rhinolophidae to investigate the feasibility of a localization mechanism that is based on processing the dominant glints. We test whether the localization cues introduced by the moving pinnae are robust against sampling. We do this by evaluating the information transfer in *R. rouxii* for a range of simulated flutter rates. We hypothesize that, the glint based localization mechanism would be feasible only if the information transfer is not hindered considerably when localization is based on the information carried by the dominant glints.

In addition, we compare the information transfer in two qualitatively different frequency channels available to the bat. In a first alternative, we analyse the information content of the response from a cochlear channel sensitive to frequencies close to the resting frequency. Such a channel produces a non-zero response throughout the duration of the echo. However, the expected response pattern, i.e. the one corresponding with the echo strength modulation due to ear movement, is disturbed by an additional unknown amplitude modulation pattern due to the fluttering prey and clutter echoes. In the other alternative, the information content of the response of a second type of channel (a Doppler shifted frequency channel) is analysed. This cochlear channel is only stimulated when large frequency shifts are introduced into the echo, i.e. during the dominant glints. We hypothesise, that using the Doppler shifted frequency channels in locating the prey will only be adaptive if its advantages (i.e. noise reduction) outweighs its potential disadvantages (i.e. information loss due to sampling).

The calls of Rhinolophidae are often preceded by a short upward sweep and/or followed by a short downward sweep. However, we only consider the constant frequency (CF) component of the calls of *R. rouxii* in our analysis. The limited bandwidth and relatively small energy in the frequency modulated (FM) component of their call has been taken to indicate that Rhinolophidae rely less on the spectral cues that are used for localization by bats emitting broadband calls [21]–[24]. Moreover, *R. rouxii* has been observed to omit the FM component in 90 percent of its calls while hanging from a perch and scanning the surroundings for flying insect prey [1], 25.

## Methods

### 3D Model of *R. rouxii* morphology

While the hearing directionality of *R. rouxii* has been measured [13], this is not the case for the emission directionality. However, simulation methods have become available that allow the evaluation of the directionality of the echolocation system of bats at a high resolution [26]–[31]. Among these simulation methods, Boundary Element Methods (BEM) are well suited to simulate both the emission and hearing directionality of bats [29]. Furthermore, BEM is thus far the only simulation method that has been formally validated for the simulation of HRTFs of small mammals (for bats [18], [29] and for gerbils [32]).

**Figure 2:**
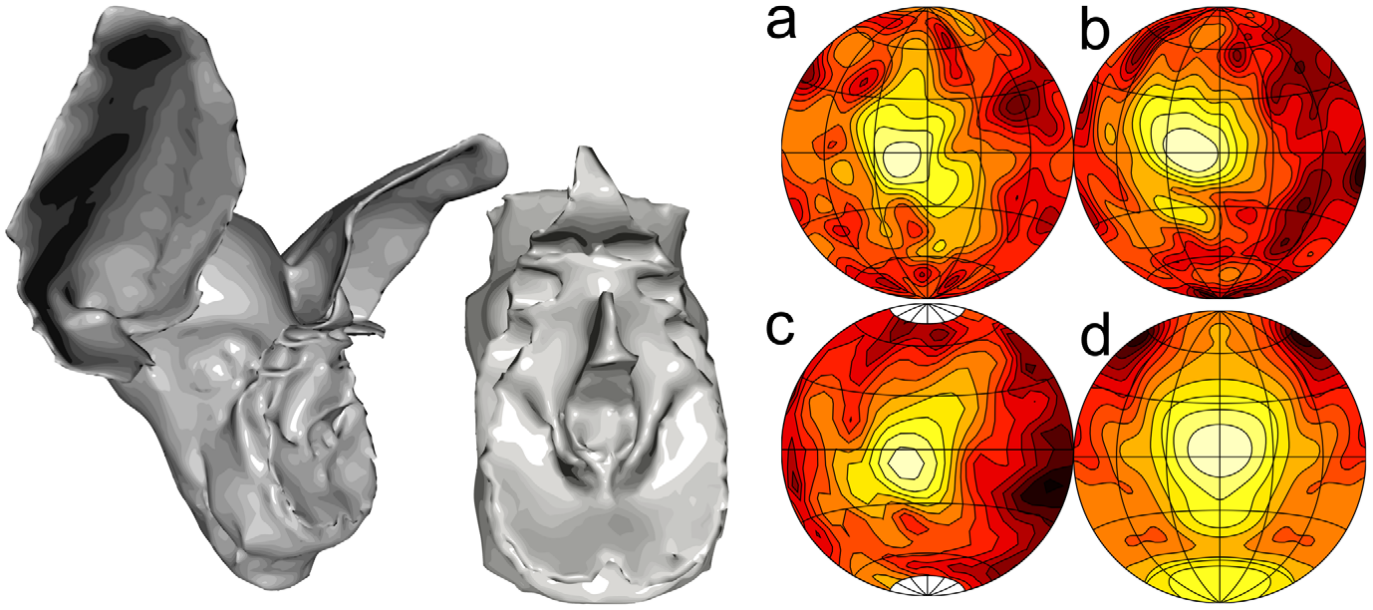
The 3D model and its simulated emission and hearing sensitivity. Left: rendering of the morphological model of *R. rouxii* and a rendering of the detailed model of the facial morphology (noseleaf) of *R. rouxii* . (a) Simulated directionality of the left ear of *R. rouxii* . The pattern was mirrored to make the comparison with ref. [13] and (c) more easy. (b) Similar as (a) but for the right ear. (c) The measured directionality as reported in ref. [13]. (d) The simulated emission pattern of the model (plotted assuming symmetry). All patterns are for 75 kHz. Contours are space 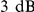 apart and depict the whole frontal hemisphere from −90 degrees to +90 degrees azimuth and elevation using a Lambert azimuthal equal-area projection. The meridians are spaced 30 degrees apart.

Using BEM to simulate the directionality of a bat requires a 3D model of the morphology of the head of the species under study. In our lab, we have developed a method to create such a model from CT data [29]. The 3D model of *R. rouxii* used in this study is rendered in figure 2. To obtain this model, a single specimen of *R. rouxii* (origin: Sri Lanka [13]) was scanned using a MicroCT machine with a resolution of 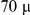
m. Using standard biomedical software and the method described in ref. [29], a 3D model of the morphology was extracted from the data (see ref. [33] for more details on the extraction of the current model).

On our current hardware, the software [26], [27] used to simulate the emission beam and the hearing directionality can only handle models consisting of up to 30,000 triangles. Therefore, we constructed a separate model of the noseleaf to ensure capturing all important features of the baroque facial morphology of *R. rouxii*. The complete head model and the model of the noseleaf are depicted in figure 2. As the resting frequency used by *R. rouxii* lies typically around 75 kHz (73–79 kHz; [1]) we use the simulated emission pattern and hearing directionality pattern at this frequency in the current paper.

Figure 2 shows the simulated hearing and emission directionality for the 3D model at 75 kHz. The simulated hearing directionality corresponds well with that reported in ref. [13]. Moreover, the match between the simulated hearing directionality of *R. rouxii* and the measured hearing directionality [13] was quantified in ref. [18] for a range of frequencies. As reported in ref. [18], we found a good agreement between the simulations and the measurements.

*R. rouxii* typically moves one of its pinnae to the front and the other one backwards during the reception of an echo. In the closely related specimen *Rhinolophus ferrumequinum*, the motion of the pinnae describe an arc of about 30 degrees at an oblique angle [15]–[17]. This is, while moving to the front (back) the pinnae also move somewhat inwards (outwards). No accurate description of the motion in *R. rouxii* is available. Therefore, we model the motion of the pinnae based on the reports on *Rhinolophus ferrumequinum* as moving from −15 degrees in elevation and −(+)15 degrees azimuth to +15 degrees in elevation and +(−)15 degrees azimuth for the right (left) ear. This is, as one ear moves down the other one moves up. In additional simulations, we confirmed that other arcs of movement influenced our results very little (see [18] for details).

Simulating the movement of the pinnae was done by assuming that this could be approximated by rigid rotations of the hearing directionality while keeping the emission directionality in the same position. In cats it has been shown that rigid rotations are a good approximation of changes to the hearing directionality due to pinnae movement [34]. Moreover, the extent over which the pinnae are moved in *R. rouxii* is rather small (about 30 degrees). Hence, we assume that the effects of the additional deformation of the pinnae on the combined emission-hearing directionality can be neglected in our analysis.

### Estimation of the Information Transfer Rate

In this section of the paper, we outline our mathematical model of the echolocation task. This model has been adapted from refs. [12], [18] and is based on the Shannon Information Theory [35], [36].

The basic assumption underlying our model is that the localization of a target can be considered as a template matching task [12], [37], [38]. A fluttering insect produces an echo containing typical target-induced Doppler shifted glints that show up as frequency spreading in the spectrogram (see refs. [4], [5], [8] for examples of spectrograms obtained from measurements). The echo is picked up by the bat's moving pinnae. Based on reports in the literature, we assume, that each pinna moves either up or down during the reception of the echo [15]–[17]. Ear movement introduces additional amplitude modulations of the echo at both tympanic membranes. The exact way in which the echo is modulated depends on the augmented head related transfer function (AHRTF), i.e., the combination of the emission directionality and the HRTF, of the bat. Each different azimuth-elevation position of a target with respect to the bat corresponds to a different expected modulation pattern at the left and the right ear. These expected modulation patterns are termed templates in the remainder of the paper. We assume the bat compares any measurement with a set of stored templates to estimate the direction from which the echo originated.

As argued in the introduction, we assume that *R. rouxii* uses the dominant glints to perform localization. Therefore, the bat has access to a version of the expected modulation patterns that is sampled at the points in time at which the echo contains a dominant glint. We assume that the bat uses a number of samples taken at discrete points in time from the amplitude modulated signal produced by the moving ears. The number of samples depends on the flutter rate of the insect. This models a worst case scenario in which a fluttering insect introduces only one dominant glint per wingbeat and the bat does not use any other glints apart from the dominant glints. Being a worst case scenario implies that the evaluation of the information transfer in Rhinolophidae using this signal results in a lower estimate. Therefore, if our results show that the frequency channels picking up the dominant glints conserve localization information, this indicates that using these channels is certainly possible for Rhinolophidae hunting under realistic circumstances where the amount of information carried by all the glints is even higher (see Discussion).

Under these assumptions, we regularly sampled the expected modulation patterns at frequencies between 20 and 200 Hz. A realistic range of flutter rates for insects as reported by [4], [5] would be about 50 to 100 Hz (see also the Discussion). Extending this range downwards enables us to assess the extent of the information transfer at very low flutter rates. Moreover, we will use the results for a flutter rate of 200 Hz as a baseline to which we compare the results for lower flutter rates. As we assume that *R. rouxii* uses calls with a duration of 50 ms [1], flutter rates of 20 to 200 Hz correspond to 1 to 10 dominant glints (samples) for each ear. The point in time of the first sample was uniformly distributed between 0 and 0.5 sampling periods. While the flutter rate of insects is very stable [9], some deviation from regularly spaced sampling are likely to occur. To investigate whether our results also hold when we no longer assume regularly spaced glints in the echo, we ran simulations in which the samples were randomly spaced over time.

The sampled versions of the expected amplitude modulation pattern at the left and the right ear are concatenated into a single vector 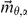 containing all measurements.

Using the same measurement noise model as proposed in [12], the received amplitudes are assumed to be corrupted both by the unknown and varying reflector strength as well as the system noise. Their different effects on the received amplitudes follow naturally if we represent the received echo amplitudes on a logarithmic scale (in 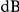), i.e., apply a compression very similar to the one performed by the hearing system. System noise is additive but, because of the logarithmic compression, its effect on the received amplitudes can be approximated by a maximum operator as,
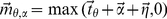
(1)

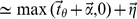
(2)with 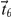 the template, i.e., the expected amplitude modulation at the different pinna positions (scaled such that 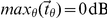), stored by the bat for reflector position 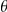. The noise level, i.e., the lower threshold below which no signal can be detected, is set at 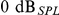. The vector 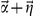 denotes the unknown and varying echo strength modulation due to the fluttering target. The term 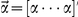 represents the mean echo strength averaged over the ear positions. As the noise level is set to zero the parameter 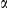 can be interpreted to specify the signal to noise ratio of the echo.

The term 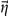 represents normally distributed multivariate noise, i.e. 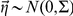 (the meaning of 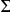 is explained in the next paragraph). This noise term models the unknown amplitude modulations imposed onto the echo due to target movement (e.g., fluttering target).

Following Bayes' theorem, the posterior probability 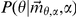 of a received vector 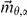 of strength 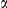 to originate from position 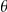 can be written as given by equation 3
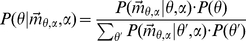
(3)Taking into account that the expected value of 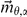, i.e., 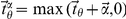, depends on 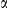, the likelihood of a received vector 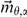 given a reflector position 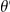 and echo strength 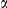 is calculated as,
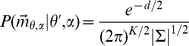
(4)with 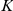 the total number of ear positions in the binaural template 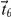 and
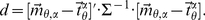
(5)The covariance matrix 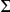 gives the variances and covariances of the stochastic vector 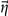. However, the amplitude of the echo 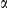 is unknown to the bat. Therefore, it is treated as a nuisance parameter in the model,
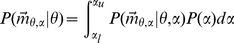
(6)with 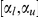 the range of 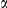 values that can occur. Hence, we rewrite equation 3 to arrive at,
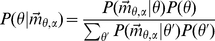
(7)


Equation 6 is calculated assuming that the bat considers all echo strengths in the interval 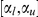 equally likely and thus maintains a uniform prior across reflector strengths. Hence, we assume that the bat has no priori knowledge about the fraction of the impinging energy reflected by the target. Equation 7 gives the posterior distribution of 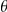. Using Shannon entropy, the uncertainty about the true target position when receiving a particular echo 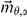 from position 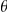 can be expressed in bits as,
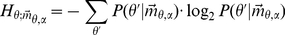
(8)


The quantity of direct behavioural relevance though is the average entropy 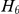 carried by all possible echoes 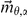 originating from position 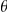. To calculate this quantity one should average over all realizations of the reflector ensemble. 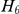 is approximated using a Monte Carlo simulation. For each position 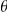, 20 realizations of the measurement vector 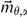 are generated. For each of these realizations, equations 3 to 8 are evaluated and the average value 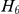 is reported. Twenty realizations for each position 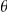 were found to yield stable results.

Having introduced the model and the methods, we can summarize all relevant assumptions: (i) localization is considered as a template matching task, (ii) we assume that only upward frequency-shifted dominant glints within a single echo are evaluated and (iii) that the relative position prey with respect to the bat does not change appreciably while it is being ensonified, (iv) it is assumed that the HRTF does not change during pinna movement, but is only rigidly rotated, (v) the parts of the echo that were Doppler-shifted by insect wings are assumed to have more stable amplitude than the echoes of non-moving objects (vi) we assume that the head does not move during call emission and echo reception (vii) FM parts of the echoes are not considered (see also discussion).

### Parametrization of the model

As described in the previous section, the model only has a single parameter, the covariance matrix 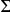,
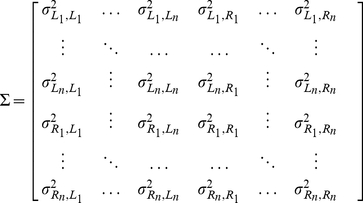
(9)with 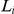 and 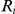 denoting the 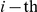 sample for the left and the right ear.

To obtain estimates of the values of 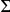 we use the value reported in ref. [4]. The authors report that the standard deviation of the amplitude of the dominant glints produced by fluttering echoes is 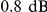 or less (see figure 5 in ref. [4]). Based on these data, we use 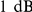 as a lower value for the diagonal of 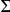.

The variation of the amplitude of the dominant glints in an echo is markedly lower than the variation of the amplitude throughout the echo as the dominant glints are, by construction, synchronized with a specific point in the wingbeat cycle. Previously, we have reported on asynchronous ensonification measurements of fluttering targets from which we calculated the standard deviation of the amplitude in a narrowband frequency channel [18]. We found a value of about 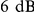. Therefore, we also evaluate the model for 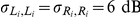. We use this value to model frequency channels that are stimulated for the entire duration of the echo signal (resulting in more noisy modulation pattern measurements) as opposed to the frequency channels that are stimulated by the dominant glints only.

Finally, in addition to 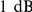 and 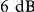 as values for the diagonal of 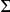, we also use 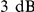 as an intermediate value to evaluate how the information transfer deteriorates when moving from a noise level of 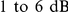.

The value of 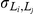, 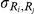 were set to 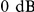. This reflects the assumption that the noise is uncorrelated across samples. Previously we found that the model is not very sensitive to the values of 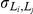 and 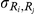 [18]. For similar reasons 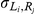 and 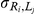 with 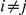 were set to 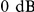 as well. Finally, 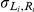 was set to 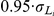 reflecting the assumption that simultaneous amplitude measurements in the left and the right ear are highly (but not perfectly) correlated (see ref. [18]).

It should be noted that 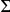 only describes the variations in the glint amplitudes within a single echo. Variations between consecutive calls are modelled as changes in the echo strength 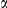. Hence, if the insect returns weaker or stronger glints across consecutive calls, this amounts to variations in the signal to noise ratio under which the bat operates.

The ability of the model to match templates and measurements critically depends on the assumed echo strength or signal to noise ratio of the echo. In the lab, fixated *R. rouxii* were found to call with an amplitude of about 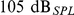 (at 10 cm in front of the bat)[39]. *R. rouxii* hunts mostly for insects with a wing length smaller than 10 mm [40]. Fluttering insects of this size return an echo that is up to 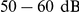 weaker than the impinging sound (depending on the frequencies used) [4]. Therefore, we evaluated the localization entropy predicted by the model for echoes ranging from 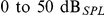 in steps of 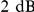 as this contains all echo strengths likely to result from prey of interest to *R. rouxii*.

In the current numerical simulations, we use 3252 templates that code for as many azimuth and elevation positions uniformly distributed over the frontal hemisphere. It was found that using more templates increased the computation time but did not alter the results. Changing the number of sample points changes the results quantitatively, but not qualitatively.

## Results

### Entropy as a function of flutter rate and noise level

**Figure 3:**
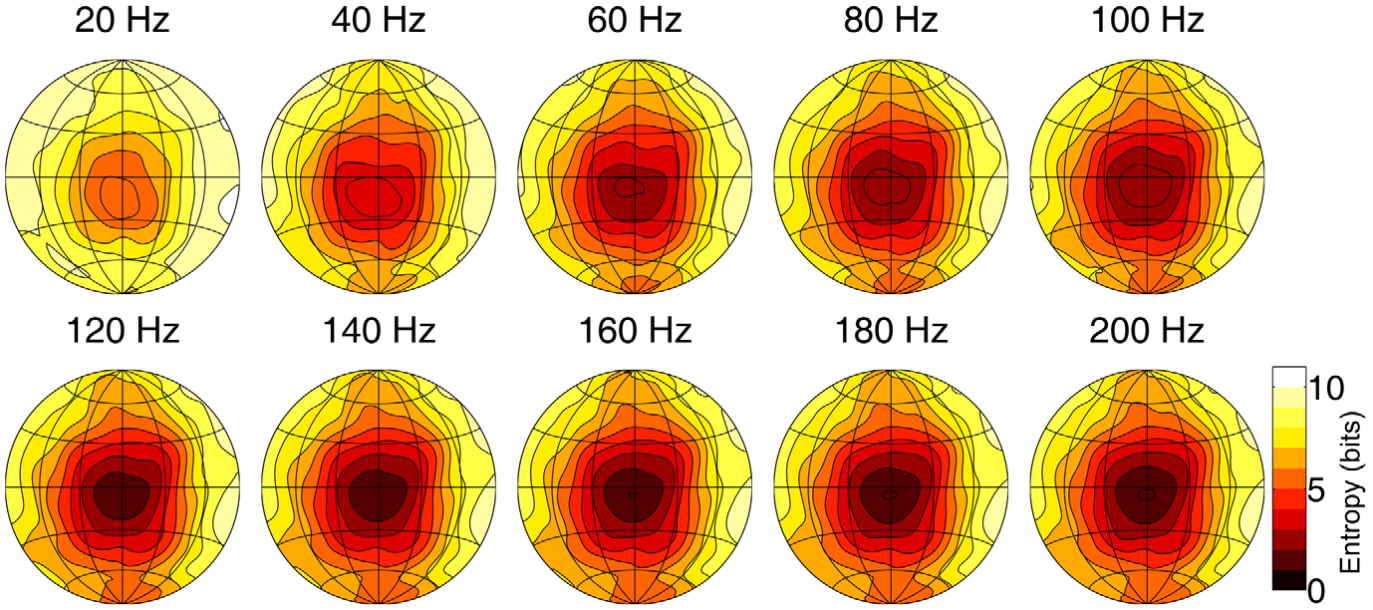
The estimated entropy about the origin of an echo as a function of azimuth and elevation. The plots depict the whole frontal hemisphere from −90 degrees to +90 degrees azimuth and elevation using a Lambert azimuthal equal-area projection. The meridians are spaced 30 degrees apart. Contour lines are spaced 1 bit apart. The simulated flutter rates are given above each panel. Higher entropy values denote lower echolocation accuracy.

The entropy, i.e., a measure of the remaining ambiguity, about the origin of an echo as function of azimuth and elevation for 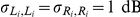 is plotted in figure 3. From this figure, it can be seen that, as the flutter rate increases, entropy quickly reaches a stable level. Increasing the flutter rate beyond 60 Hz does not reduce entropy significantly. It should also be noted that at 20 Hz, the lowest flutter rate simulated, the predicted echolocation entropy is already considerably lower than chance level (i.e. about 11 bits in the current simulations). In a central area, entropy goes down to a level of about 6 bits even for this low sampling frequency. Note that, at a flutter rate of 20 Hz, the model is only provided with 1 sample per ear to perform localization.

**Figure 4:**
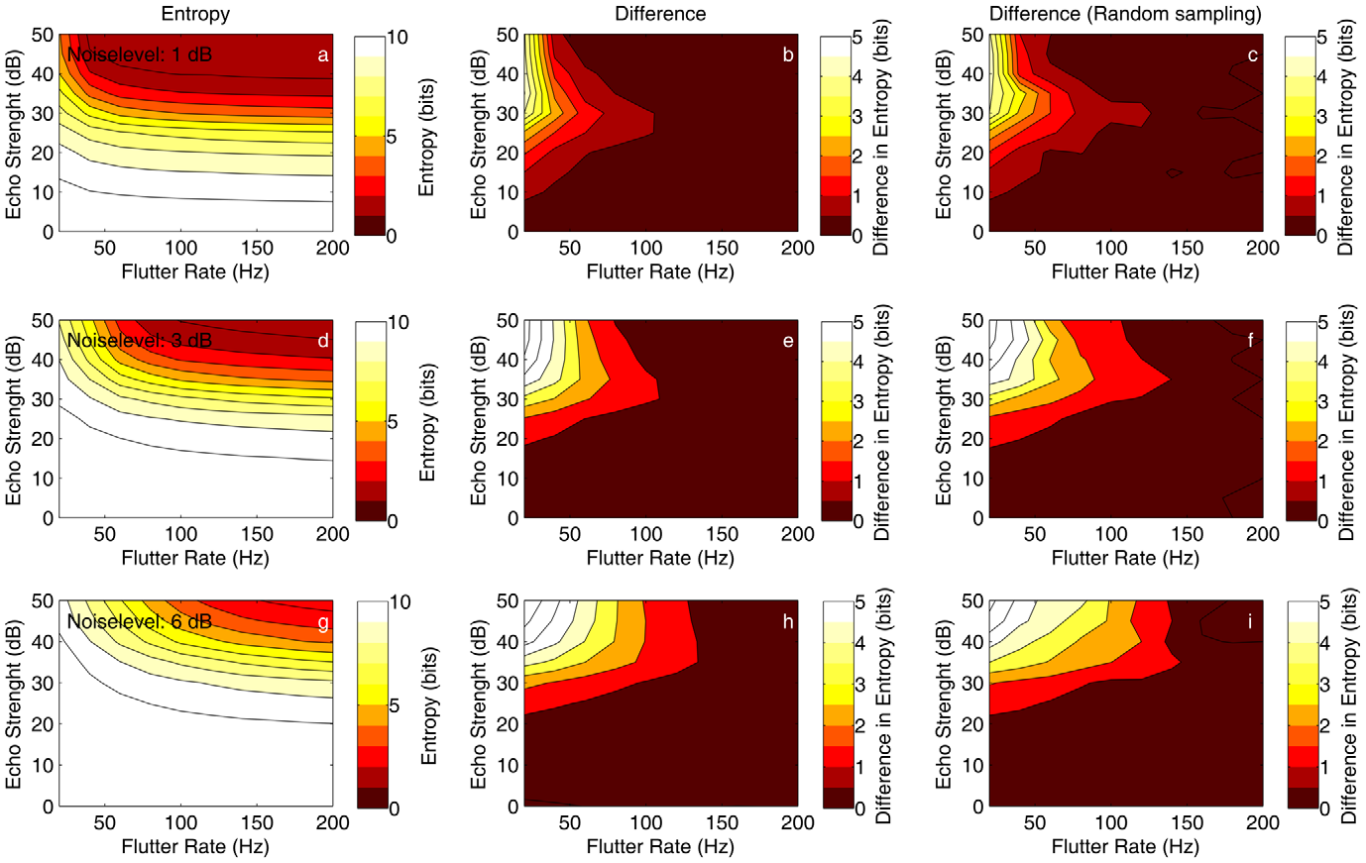
The effects of sampling and higher noise levels on the information transfer. The different rows represent higher noise levels (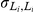 and 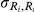). The first column (a, d & g) shows the average entropy (across azimuth-elevation positions) as a function of flutter rate and echo strengths. The second column (b, e & h) shows the difference in entropy between each flutter rate and the entropy for a flutter rate of 200 Hz. Therefore, these plots illustrate the net effect of having less samples on which to base localization. The third column (c, f & i) is similar as the second one but was based on simulations in which samples were randomly spaced in time.

Figure 4 further explores the effect of flutter rate on localization entropy. Figure 4 confirms that entropy mostly depends on the echo strength. For every flutter rate the entropy decreases as echo strength increases. In contrast, entropy depends only little on flutter rate as long as the flutter rate is higher than about 50 Hz when 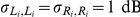 (fig. 4b & c). Indeed, for these flutter rates the difference in entropy between the information transfer at a flutter rate of 200 Hz and a lower flutter rate is less than 1 bit. For higher noise levels, the overall entropy increases (fig. 4a, d & g). Moreover, the effect of flutter rate increases with increasing noise levels (fig. 4b, e & h). However, even for 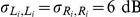, the effect of flutter rate is mainly located in the region of flutter rates lower than 100 Hz.

The third column of plots in figure 4 confirms that the results also hold when the echo is sampled at random intervals (in contrast to fixed intervals). This indicates that our results are not sensitive to a deviation from regular spaced sampling.

**Figure 5:**
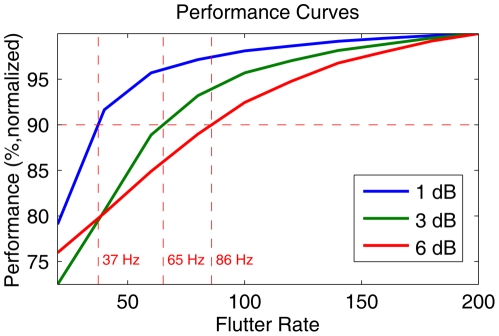
Performance curves for the 3 noise levels in percentages. In this plot, a performance of 100% is the entropy level for a flutter rate of 200 Hz. The performance figures have been calculated on the averaged entropy levels for all echo strenghts. The flutter rates at which 90% performance is attained for the three noise levels are indicated.

Another way of summarizing the information loss due to sampling is given in the performance plots in figure 5. The performance 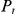 measure plotted in this figure for a given flutter rate 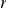 is calculated as follows,
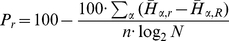
(10)with 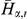 the entropy for a given flutter rate 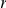 and echo strength 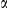 (averaged across the frontal hemisphere). Flutter rate 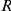 is the highest flutter rate simulated, i.e. 200 Hz. The parameters 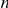 and 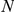 give the number of echo strengths evaluated and the number of templates (i.e. 3252) respectively. Therefore, in plot 5, a performance of 100% is the entropy level for a flutter rate of 200 Hz and the plot shows the normalized average performance as a function of flutter rate for the three noise levels. It can be seen that 90% performance is reached for the three noise levels at flutter rates 37, 65 and 86 Hz respectively.

The results presented so far indicate that, for the noise levels typical for the dominant glints (i.e. about 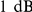), the localization entropy does not depend heavily on the flutter rate of the insect that is to be located. The results plotted in figures 4 and 5 indicate that *R. rouxii* looses little performance by sampling the echo even when the flutter rate is low. In addition, these results indicate that the robustness against sampling is higher for lower noise levels. The dependence on flutter rate increases gradually as the noise level rises.

### Comparing the information transfer for two different types of frequency channels

**Figure 6:**
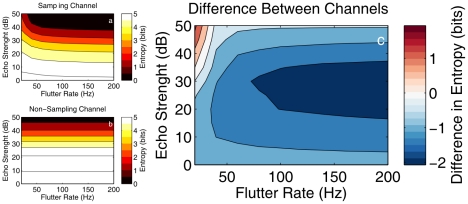
Comparison of the entropy for the two frequency channels. (a) Localization entropy as a function of flutter rate and echo strength for a frequency channel that is stimulated only by the most Doppler-shifted parts of the dominant glints (sampling, but low noise level, i.e. 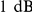); (b) localization entropy as a function of flutter rate and echo strength for a frequency channel that responds for the entire duration of the echo (no sampling, but high noise level, i.e. 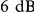). Note that, in panel (b) the entropy does not depend on the flutter rate as no sampling at the dominant glints is performed. (c) The difference between (a) and (b).

In figure 6, the entropy for the two types of frequency channels described in the introduction are compared.

As can be seen in figure 6, the information content of the output of the frequency channel responding for the entire duration of the echo signal but suffering from a higher noise level is almost uniformly the lowest. Indeed, for almost every flutter rate and echo strength the entropy about the location of a target is higher for the ‘non-sampling’ frequency channel than for the ‘sampling’ frequency channel. Only for very low flutter rates and very high echo strengths are the roles reversed. This indicates that, although some information is lost due to sampling the echo signal, the noise reduction that is achieved by processing only the most Doppler-shifted parts of the dominant glints yields an almost universal increase in target location information.

### Template robustness in the presence of sampling

In theory, there are two ways in which templates can be robust against sampling. First, templates could show a high degree of variation. By having templates that have a higher dynamic range, the Euclidean distance between templates increases and any loss in fidelity by sampling would cause less increase in localization entropy. Alternatively, templates could have most of their energy in the lower frequency components of the modulation spectrum. In this case, the spectrum of a template would only contain low frequency components. If templates would only vary slowly as the pinnae move through space, any sub-sampling would be less of a problem. It should be noted that these two strategies to design more robust templates are somewhat contradictory. Templates that have a larger dynamic range will usually contain higher frequencies.

**Figure 7:**
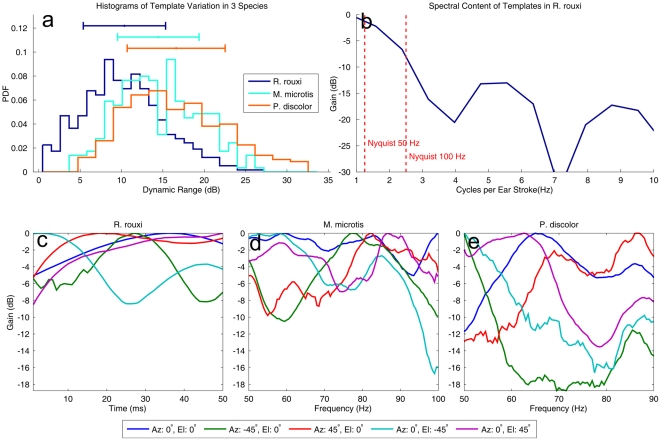
Properties of the templates of *R. rouxii* , *M. microtis* and *P. discolor*. (a) Histograms of the dynamic range of the templates of three species of bats. *M. microtis* and *P. discolor* are FM bats that, in contrast to *R. rouxii* , localize targets by means of spectral cues provided by their broadband calls. The horizontal lines denote the −1 and +1 standard deviation intervals. (b) The average spectrum of the templates of *R. rouxii*. The frequency scale is expressed as the number of cycles per ear stroke, i.e. one forward or backward sweep of the pinna. (c–e) Illustration of 5 templates of *R. rouxii* , *M. microtis* and *P. discolor* . The locations for which the templates code are indicated by their line colour. Note that the x-axis of the templates for *M. microtis* and *P. discolor* is expressed in kHz. The frequency ranges plotted are the ones relevant for these two bats.

In figure 7a & b, we plotted a histogram of the dynamic range of the templates of *R. rouxii* and an average spectrum of the templates respectively. In figure 7a, the dynamic range of the templates of *R. rouxii* is compared with those of *Micronycteris microtis* and *Phyllostomus discolor*. We have previously reported on the simulated HRTFs and emission patterns of these bats [30]. Moreover, we have provided an analysis of the localization information transfer of *M. microtis* [12]. In contrast to *R. rouxii* , both *M. microtis* and *P. discolor* emit short broadband calls and use spectral cues as means of localizing echoes in space. In these animals, as in most mammals, the major part of the localization information is provided by notches in the spectra generated by the filtering of the pinnae [12], [41]. Therefore, in contrast to *R. rouxii* which is assumed to use amplitude modulations of a narrowband signal, these bats mainly code the position of a target in space by means of spectral notches.

In figure 7a, it can be seen that the dynamic range of the templates of *R. rouxii* is not larger than that of the two other bats. Inspecting some examples of the templates of the three species (plotted in figures 7c–e) it can be seen that the templates of *R. rouxii* do not show the deep notches found in *M. microtis* and *P. discolor*.

The templates of *R. rouxii* , consist mostly of low frequency components (figure 7b). However, a major part of the energy is contained in frequency components for which the Nyquist criterion is not reached at typical flutter rates of insect prey. For example, targets fluttering at 100 Hz yield 5 glints in an echo of 50 ms. This only allows to faithfully reconstruct frequencies up to 2.5 Hz (see line in figure 7b). Stated differently, reconstructing the templates from the samples provided by a target that flutters at 50 Hz is only possible if the templates contained only frequencies below 1.25 Hz.

In sum, the templates of the echolocation system of *R. rouxii* do not seem to be particularly suited to be robust against sampling at the rates their prey flutters. Neither the dynamic range nor the spectra of the templates seem optimized for reconstruction from a small number of samples. Hence, we propose that the localization system of *R. rouxii* is robust against sampling of the templates only because it can effectively limit the noise by processing the dominant glints. Indeed, the results plotted in figure 4 and 5 indicate that good localization for low flutter rates is only attained if the noise level is low.

## Discussion

Our simulation results show that Rhinolophidae could reject unwanted amplitude variations (i.e. noise caused both by clutter and target movement) by processing dominant glints without substantially reducing the localization information transfer. Plots 1a–c show that almost no localization information is lost once the flutter rate is higher than about 50–60 Hz (for a noise level of 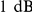). Indeed, the performance curves in figure 5 show that a performance level of 90% is attained at a flutter rate of about 40 Hz.

Although some insects have flutter rates even below 20 Hz (the lowest flutter rate simulated) [42], a flutter rate of 40 Hz seems in the lower range of the flutter rates frequently encountered by these echolocating bats [4]. Since no data exists, as far as we could find, about the distribution of the flutter rates of insects *R. rouxii* preys on it is unknown what range of flutter rates is of behavioural importance to the bat. However, indirect evidence, i.e. cortical neurons that encode flutter rates show best phase locking for flutter rates between 40–60 Hz in *R. ferrumequinum*, seems to indicate that flutter rates in the range 40–60 Hz might indeed be relevant to *R. rouxii* . Moreover, many insects have flutter rates in this range (see [3] for references). Furthermore, by lengthening its emissions, a simple adaptive strategy Rhinolophidae makes use of when faced with a difficult echolocation task [1], *R. rouxii* could locate insects with lower flutter rates. Our simulations are based on a call duration of 50 ms. Doubling the length of the call would imply that the simulated flutter rates could be halved without altering the results. In addition, we have assumed that insects produce a single dominant glint per wing beat. However, depending on the wing structure, some insects produce more than a single dominant glint per wing beat cycle [4]. Insects that produce multiple dominant glints would provide the bat with more localization information and should therefore be easier to locate at lower flutter rates. The fact that Rhinolophidae can lengthen their call and that some insects produce multiple dominant glints increases the feasibility of using channels sensitive to the Doppler shifted dominant glints.

More important than the absolute information transfer for any channel is the comparison between the two types of channels proposed in the introduction. We compared the information transfer in both types of channels in figure 6. It was found that Doppler shifted frequency channels almost invariably outperform the channels responding to the centre frequency.

While our simulations show that Doppler shifted channels provide Rhinolophidae with better localization performance than the more noisy channels responding to the reference frequency, bats will have access to both types of channels while locating prey. Indeed, bats can support the information in the Doppler shifted frequency channels with information gathered by reference frequency channels. Therefore, our simulations yield a conservative, i.e. lower bound of the information transfer, and real bats likely use both the reference frequency as well as the frequency shifted parts of the echo, as would be expected from their sensory physiology.

Neurophysiology recordings in the cochlear nucleus suggest that Rhinolophidae posses neurons that can support the processing of self-induced modulations of the dominant glints in the echoes. Their cochlear nucleus contains a large proportion of neurons with a high degree of frequency tuning that respond only to the onset of the preferred frequency [43]. About 40% of these neurons were found to be insensitive to variations in intensity. These neurons would be well suited to detect dominant glints in the echoes. A tentative hypothesis about the implementation of the glint based localization proposed in the current paper could be as follows: neurons selective to frequency and with a phasic response continuously monitor Doppler shifted frequency channels. These onset-coding and intensity-insensitive neurons act as a clock pulse selecting samples from the continuous intensity signals coded by other intensity-sensitive neurons.

In addition, it should be noted that, although 40% of the onset-coding neurons in the cochlear nucleus were found to be insensitive to intensity [43] at least some neurons in the cochlear nucleus are capable of detecting both the onset and encoding the intensity (by means of prolonged firing [43]). Also, similar properties of sharp frequency tuning and amplitude modulation selectivity can be found in other auditory nuclei in rhinolophid bats, e.g. [44], [45]. This indicates that the localization mechanism proposed in this paper could be implemented at different levels in the auditory system of Rhinolophidae.

Finding that the localization information transfer in *R. rouxii* is robust against sampling, we analysed the templates in order to investigate whether these show any adaptations that support this robustness. However, we could find no evidence of the templates of *R. rouxii* showing adaptations to being sampled at the flutter rates of likely targets. On the contrary, on average the templates show less dynamic range than those used by *M. microtis* and *P. discolor* . Also, the sample rate dictated by the flutter rate of insects is not high enough to comply with the Nyquist criterion as the templates contain frequency components that are too high. Rather, it seems that the reduction in echo amplitude variability (i.e. noise) by focusing on dominant glints allows the bat to locate targets without such adaptations.

**Figure 8:**
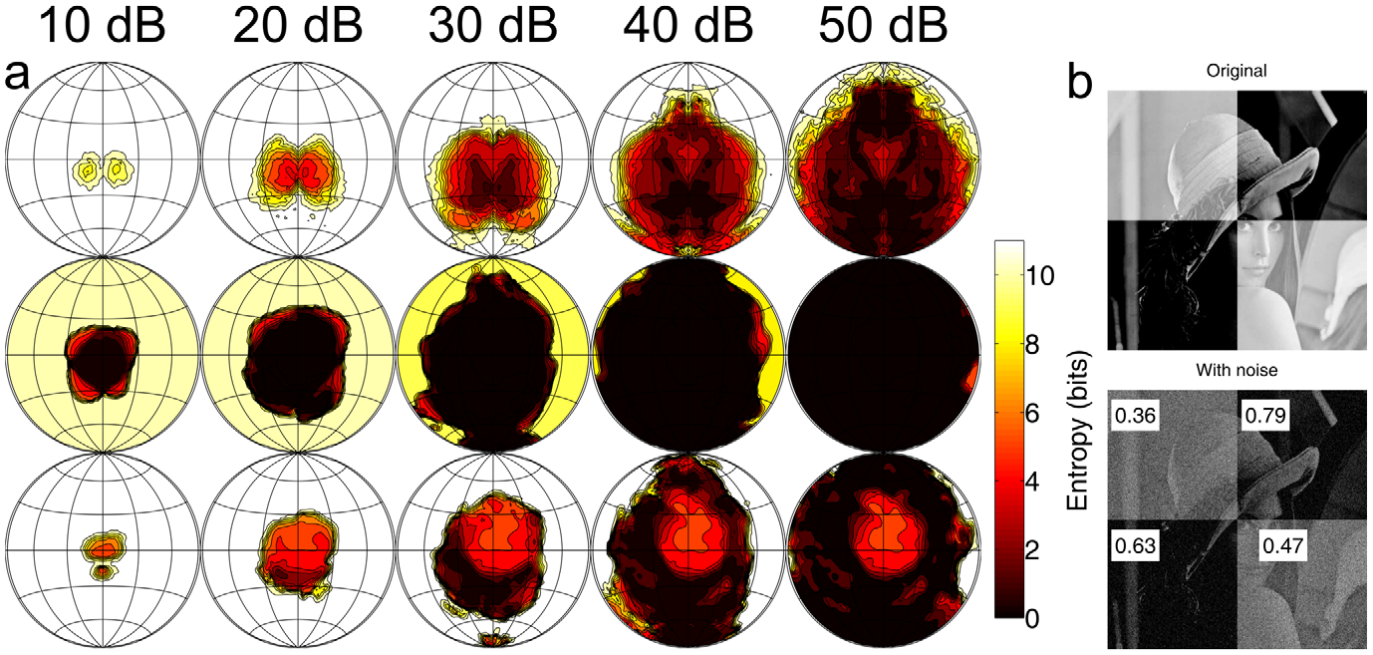
Illustration of the sensitivity-accuracy trade-off. (a) Top row: Localization entropy plots for *M. microtis* as a function of echo strength (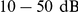) and azimuth-elevation position. The entropy in the centre, i.e. the most sensitive region, reaches a minimum around echo strength of 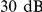. For echo strengths above 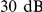 entropy in the periphery (i.c. low elevation positions) is lower than around 0 degrees azimuth and elevation. Middle row: Plots of the localization entropy of *R. rouxii* as a function of echo strength (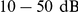) and azimuth-elevation position (noise level 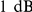). The plots are for a flutter rate of 200 Hz. These plots show that the localization entropy in *R. rouxii* is always lowest in a central region and that the region of low entropy expands as a function of echo strength. Bottom row: Similar as the middle row but for a noise level of 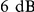. (b) Visual analogue to illustrate the sensitivity-accuracy trade-off. The top left and the bottom right quadrant of the classic Lenna image [46] were given an increased intensity. The quadrants serve as an analogue to the highly sensitive templates found in the central region of the echolocation systems of bats. The other two quadrants were given an increased contrast, i.e. as an analogue to the peripheral templates with a higher dynamic range. In the bottom figure the top figure has been corrupted by additive noise. The correlation between each quadrant in the top and the bottom figure is indicated by the numbers in the bottom figure. It can be seen that the quadrants with the highest contrast (i.e. the lowest average intensity or sensitivity) have the highest correlation. This indicates that high contrast images, while having a lower average intensity, are less affected by noise. Similarly, localization templates with a high dynamic range are less disturbed by noise but render the bat less sensitive.

Analysing the localization information transfer of the FM bat *M. microtis* it was found that the spectral templates with the largest dynamic range encode peripheral positions. Notches in the spectral templates of this bat are mostly found for peripheral positions. However, these notches, being created by side lobes in the spatial sensitivity pattern of the system, lower the sensitivity of the system at these locations. Indeed, deep notches in a template denote a combination of a location and a frequency for which the system is insensitive. The effect of this on localization is that weak echoes can be located best in a central region where sensitivity is highest. However, for stronger echoes the region with the best localization is actually the periphery at locations coded by deep spectral notches as these templates are more resistant to unknown reflector filtering (noise), see figure 8 and [12]. Therefore, in the face of noise, these bats are confronted with a trade-off: for any given position the bat can either be highly sensitive or very accurate.

The switch in the region where echoes can be best located, is not observed in *R. rouxii* . Plotting the localization entropy as a function of echo strength (see figure 8) it is found that lowest entropy is always located in the central region and that this region of low entropy simply expands as the echo strength increases. By focussing on the dominant glints, the entropy in the central region is not increased by noise and the bat does not experience a trade-off. Indeed, figure 8 shows that avoiding the trade-off is only possible for low noise levels (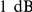). For a noise level of 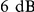, entropy in the central region is also higher than for the peripheral region in *R. rouxii* . In this case, *R. rouxii* show the same trade-off as *M. microtis* and, because of the overall lower dynamic range of its templates, the trade-off is even more pronounced as can be seen from the larger contrast between its central and peripheral localization entropy (bottom row of figure 8). This figure also contains a visual analogue using the classic Lenna image [46] to further clarify the sensitivity-accuracy trade-off faced by most bats.

Interestingly, in addition to theoretical evidence for this fundamental trade-off [12], direct behavioural evidence was recently found. The bat *Rousettus aegyptiacus* was shown to point its beam not directly towards a target it needs to localize but slightly to the left and to the right of it. Hence, it receives less energetic but more informative echoes from the object of interest [47], thereby trading sensitivity for accuracy.

Concluding, we propose that the dominant glints, showing little amplitude variations (i.e. noise), are ideal input signals for a system using self-induced amplitude modulations to locate targets. Indeed, the low noise levels attained by only processing dominant glints, outweighs the loss in information due to sampling of the echo.
